# Comparative Study of Clinical Features of Patients with Different Types of Benign Paroxysmal Positional Vertigo

**DOI:** 10.3390/jcm13164736

**Published:** 2024-08-12

**Authors:** Marlena Ziemska-Gorczyca, Karolina Dżaman, Dana Pavlovschi, Ireneusz Kantor, Andrzej Wojdas

**Affiliations:** 1 Department of Otolaryngology, Centre of Postgraduate Medical Education, Marymoncka 99/103, 01-813 Warsaw, Poland; marlena.z.gorczyca@gmail.com (M.Z.-G.); pawlowsky.dana@gmail.com (D.P.); ireneusz.kantor@gmail.com (I.K.); 2 Department of Otolaryngology, Military Institute of Aviation Medicine, Krasińskiego 54/56, 01-755 Warsaw, Poland; awojdas@wiml.waw.pl

**Keywords:** vertigo, dizziness, BPPV, apogeotropic nystagmus, semicircular canals

## Abstract

**Objectives**: Even though BPPV is one of the most common causes of vertigo, it is often underdiagnosed and omitted in the diagnosis of patients reporting vertigo. The aim of the study was to establish a diagnostic pattern useful in patients admitted due to vertigo, based on the most common clinical characteristics of patients suffered from posterior canal BPPV (PC-BPPV), horizontal canal BPPV with geotropic (HCG-BPPV) and apogeotropic nystagmus (HCA-BPPV). **Methods**: The analysis covered the results obtained in 105 patients with a positive result of the Dix-Hallpike maneuver or the supine roll test. The patients were divided into 3 groups based on the BPPV type: gr.1:PC-BPPV (60%); gr.2: HCG-BPPV (27%); gr.3: HCA-BPPV (13%). Patients before the diagnostic maneuvers filled the questionnaire concerning their symptoms and previous diseases. **Results**: Almost all patients had vertigo during turning over in bed and the character of the symptoms was paroxysmal. The answers to questions about the type of head movement evoked vertigo and how long vertigo lasted were differentiating. The percentages of correct diagnosis speculated by the combined answers were 69.6% in PC-BPPV, 61.8% in HCG-BPPV, and 80% in HCA-BPPV. **Conclusions**: Basing on those observations there is presented the diagnostic schedule which could be useful in dizziness examination. The above results indicate that a properly collected interview with the patient allows for a high percentage of accurate diagnosis.

## 1. Introduction

Patients with dizziness pose a major diagnostic challenge for the physicians. Dizziness, vertigo and imbalance account for 1–2% of consultations among outpatients in general practice [1]. Dizziness is a symptom which can be described by patients in various ways, such as a false sense of motion, spinning (vertigo), lightheadedness, feeling faint, unsteadiness, loss of balance, feeling of floating, wooziness or heavy-headedness. Vertigo is a sensation of spinning, tilting, or that one’s surroundings are rotating, it might be described by patients also as dizziness which might be misleading for physicians. In-depth interview with a patient often allows to make an accurate diagnosis. Benign Paroxysmal Positional Vertigo (BPPV) is one of the most common causes of vertigo. BPPV is characterized by short-term attacks of spin-like dizziness with accompanying nystagmus, triggered by a change in the position of the head relative to gravitational forces. Vertigo attacks may be accompanied by a feeling of disorientation in space and a feeling of “dizziness”, periodic nausea, drenching sweats, a feeling of fear, rarely vomiting and headaches. BPPV affects people of both sexes and all ages (including children). A significant increase in the incidence is observed in people over 45 years of age. The incidence rate at the age of 18–39 is 0.5%, and over 60 years old–3.4% [2]. BPPV affects women more often than men (2–3:1) [3]. The lifetime prevalence of the disease is estimated at 2.4% in the general population [2]. About 1.6% of new cases are diagnosed annually [2]. Untreated BPPV increases the risk of falls, reduces daily physical activity and increases the risk of developing depression, which leads to a decrease in quality of life [4]. Additionally, in people over 65 years, the coexistence of BPPV with other diseases such as: arthritis, degenerative changes in the musculoskeletal system, neuropathies, orthostatic hypotension, vision disorders, etc., significantly increase the risk of falls and their consequences. Based on the consensus of the Classification Committee of Vestibular Disorders of the Bárány Society, diagnosing BPPV relies on performing diagnostic maneuvers and conducting a thorough patient interview [5]. In the previous studies investigators designed a questionnaire which helps to diagnose BPPV with sensitivity 81% and the specificity 69% [6]. It is worth mentioning that performing diagnostic maneuvers with the removal of fixation reduces the risk of false negative results, especially in the case of fatigue of nystagmus.

The posterior semicircular canal is the most commonly affected in BPPV, with posterior canalolithiasis accounting for about 60–90% of all cases [3,5]. The lateral semicircular canal is the second most frequently involved, with canalolithiasis and cupulolithiasis representing 10–20% of cases [7,8]. Other forms, such as posterior semicircular cupulolithiasis, anterior semicircular canalolithiasis, and both unilateral and bilateral multicanal BPPV, are relatively rare. Different types of diseases process e.g., osteoporosis, high total cholesterol level, migraine, and head trauma favor the development of otolith disorders [9]. The resulting disorders contribute to the release of otoliths from the otolith membrane of the utricle, and different positions of the head enable their migration to non-physiological places such as the semicircular canals. During the movement of the head, the displacement of the otoliths causes the generation of pathological active potentials within the receptors in cupula, which results in the feeling of vertigo. Because the cupula is an impermeable barrier to the otoliths, these deposits are “trapped” in the duct and can only exit through the non-ampular branch of the canal.

Currently, there are three theories explaining the pathomechanism of BPPV: the cupulolithiasis theory, the “canalith jam” theory and the canalolithiasis theory. The theory of canalolithiasis was proposed over 40 years ago by Hall et al. and then confirmed by Epley [5,6]. Freely moving otoliths in the lumen of the semicircular duct cause a deflection of the cupula, which is achieved as a result of the “piston effect” (otoliths compress the endolymph in front of them, pushing it forward, and behind them a zone of reduced pressure is created) or as a result of hydrodynamic resistance (the hydrodynamic force component is directed in the opposite direction to the otolith movement) [10]. Triggered nystagmus in the mechanism of canal stones is characterized by latency, sudden onset, gradual extinction and fatigue.

Canalith jam is an uncommon ailment that arises when a cluster of otoliths clogs a slim segment of the membranous duct, obstructing the flow of endolymph. This situation creates a constant change in hydrostatic pressure between the otoliths aggregation and the cupula, resulting in an ongoing bending the cupula [11].

The theory of cupulolithiasis is still debated. According to the theory of cupulolithiasis, pathological deposits are deposited on the surface of the cupula. By deflecting it, they cause attack of vertigo. Recently, the metanalysis concerning the cupulolithiasis theory has appeared, the authors stated that only around 31% of apogeotropic nystagmus in horizontal canal BPPV may be caused by cupulolithiasis or canalith jam [12]. The most often periampullary canalolithiasis causes the apogeotropic BPPV [12]. Further studies are needed in this case. 

The aim of the study was to establish a diagnostic pattern useful in patients admitted due to vertigo, based on the most common clinical characteristics of patients suffered from posterior canal BPPV (PC-BPPV), horizontal canal BPPV with geotropic (HCG-BPPV) and apogeotropic nystagmus (HCA-BPPV). 

## 2. Materials and Methods

The prospective study enrolled 211 patients suffered from paroxysmal vertigo and balance disorders with characteristic clinical features for BPPV. The patients were treated at the Department of Otolaryngology of the Mazovian Bródno Hospital in Warsaw in 2018–2020. Based on the consensus of the Classification Committee of Vestibular Disorders of the Bárány Society, 105 of patients have been subjected to further analysis. Patients filled out a questionnaire and were investigated for BPPV with diagnostic maneuvers- the Dix-Hallpike maneuver (D-H maneuver) and the supine roll test. In patients classified to the PC-BPPV group, during the D-H maneuver, there was observed a characteristic nystagmus, which was a combination of rotational nystagmus beating towards the lower (affected) ear with vertical component beating towards the upper eyelid. In HCG-BPPV group, the supine roll test revealed geotropic horizontal nystagmus, and the side with stronger nystagmus was the affected side. In HCA-BPPV group apogeotropic horizontal nystagmus was induced and the side with weaker nystagmus was the affected side. The survey concerned patient’s chronic diseases, past medical history, frequency of attacks and the presence of vomiting and nausea during attack (Table 1).

**Figure 1:**
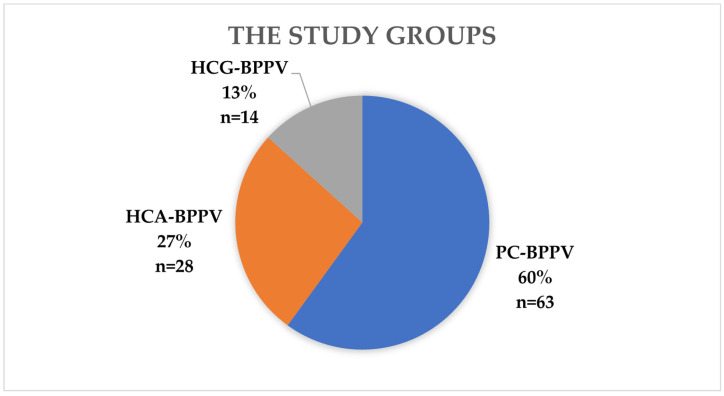
The presentation of the study groups. Legend: HCG-BPPV—horizontal canal benign paroxysmal positional vertigo with geotropic nystagmus; HCA-BPPV—horizontal canal benign paroxysmal positional vertigo with apogeotropic nystagmus; PC-BPPV—posterior canal benign paroxysmal positional vertigo.

Patients were asked about any past head injuries and whether there were any lasting consequences from those injuries. There were no condition concerning the severity of head injury. Patients were questioned if the vertigo appeared during turning in bed, looking up or down (movement of their head in sagittal plane), turning head side to side (movement in horizontal (axial) plane), changing the body position. for questionnaire. All 105 patients included in further analyses were divided as follows into 3 groups based on the type of BPPV (Figure 1).

The first study group consisted of 63 patients (60.0%) who were diagnosed with PC-BPPV. The patients diagnosed with HCG-BPPV (26.7%; *n* = 28) were included in the second study group. The third study group consisted of 14 patients (13.3%) diagnosed with HCA-BPPV. The D-H maneuver and the supine roll test were performed in all patients. Diagnostic maneuvers were performed manually according to generally accepted assumptions [13,14]. The presence of triggered nystagmus as a positive test result was recorded using the Frami-VCOR device and video googles (allowing the registration of nystagmus) by FRAMIRAL. The obtained results were analyzed statistically: a descriptive statistics, the Shapiro–Wilk test, hypothesis testing using the nonparametric modified chi-square test, post-hoc analysis and the parametric test the repeated-measures Student’s *t*-test. The specificity and sensitivity calculations were conducted to assess the usefulness specific questions during the examination of patient with vertigo. Sensitivity measured the proportion of actual positives that were correctly identified by the combined answers in the questionnaire. This metric reflects the ability of the test to correctly identify individuals with the specific type of BPPV. Specificity measures the proportion of actual negatives that are correctly identified by the test. This metric reflects the ability of the test to correctly identify individuals without the specific type of BPPV. In our study, the performance of the diagnostic test was evaluated on a sample of 105 individuals. The statistical tests were performed in R statistical software (version 4.2.3).

## 3. Results

The study groups did not differ in age and sex structure. The mean age was 47.7 years among all study groups. There were no statistically significant differences in the history of head injury (*p*-value 0.257), the presence of migraine (*p*-value 0.143) and hypertension (*p*-value 0.337) among all study groups.

None of the patients who reported a head injury had any lasting sequelae of the injury. Patients in HCA-BPPV group the most often reported head injury and migraine (35.7%), as well as hypertension (42.9%). In the entire study population migraine was reported by 20%, hypertension by 27.6% and head injury by 24.8%. In all groups the right ear was more often affected than the left one (in PC-BPPV–60.3%, in HCG-BPPV–67.9%, in HCA-BPPV–57.1%, overall–61.9%) and there were no significant differences between the study groups regarding the side of the affected ear. 

The first episode of BPPV was reported by 46.7% study participants (n = 49). The rest patients (53.3%) had similar symptoms before but they did not have any diagnostic maneuvers when the vertigo appeared. Patients with the first episode of BPPV the most often had symptoms lasting 1 to 7 days. Patients with recurrent episode of BPPV more often had the episode lasting more than one month (66.0%). 

The characteristics of patients from the three study groups differed both in terms of the description of the dizziness itself and the accompanying symptoms. Among the study groups statistically significant differences were observed in: the feeling of the undulation of the ground, presence of nausea and vomiting during the attacks, the frequency of attacks, the duration of the attacks, evoked vertigo by turning in bed, movement head in sagittal and horizontal planes. The results are presented in Table 2. 

Attacks of vertigo occurred more than once a day in half of the patients in HCG-BPPV group which was significantly more frequent than in other groups. A similar frequency was reported by only 14% of patients with HCA-BPPV and every third patient with PC-BPPV. In PC-BPPV group nearly 50% of patients had attacks once a day, and the lowest frequency of BPPV attacks was in HCA-BPPV where 42.5% of patients reported that attacks occurred a couple times a week. The length of the vertigo episode varied significantly depending on the group. Short–lasting attacks of vertigo which lasted a couple of seconds predominated in the PC-BPPV group and occurred in half of the patients with HCG-BPPV. In the HCA-BPPV group, the duration of vertigo more often exceeded 1 min (28.6% of patients) than in other groups (HCG-BPPV 3.6%, PC-BPPV 0%). The character of vertigo was paroxysmal in all patients among all study groups. Severe vegetative symptoms were observed in the majority of patients with HCG-BPPV (64.3% nausea, 32.1% vomiting). Only every third patient in PC-BPPV group presented with vegetative symptoms, and most often they were of a milder nature (nausea, not vomiting). Vegetative lesions were not recorded in any patient in HCA-BPPV group. An additional element that may influence the incidence of nausea and the duration of attacks is the activity of individual patients during the day. More active people might experience more ailments.

In the vast majority of patients of all groups, the factor causing vertigo was turning in bed. A change in the head position side to side provoked an attack in the majority (75%) of patients with HCG-BPPV which was statistically significantly more often than in the other groups.

Moreover, we compiled the most popular answers to given questions for a given type of BPPV, then we performed sensitivity and specificity calculations. The results of these calculations are presented in the Table 3. 

The percentage of patients with PC-BPPV who had vertigo after head movement in the sagittal plane which lasted couple of seconds was 61.9% and was significantly higher than patients with both HCG- and HCA-BPPV (40.5%) (*p* < 0.05). Moreover, the specificity of the above mentioned combined answers is relatively low- 51%, it means that 51% patients with HCG or HCA BPPV will be misdiagnosed with PC-BPPV. It might be a result that relatively high percentage of patients with horizontal canal BPPV have symptoms during head movement in sagittal plane. The combine answers that vertigo was evoked by movement of the head in the axial plane and it lasted less than one minute had high specificity for patients diagnosed with HCG-BPPV (86.3%). This suggests that the combined answers correctly identified 86.3% of those without the HCG-BPPV (true negative rate). The lower sensitivity means that 61.8% of patients with HCG-BPPV will be correctly diagnosed. The other significant observation was that the ratio of patients with HCA-BPPV who answered that the duration time of vertigo was more than 1 min among all patients with HCA-BPPV (40%) was four times higher than in other types of BPPV (1%) (*p* < 0.05). As a consequence, the high specificity (90%) and sensitivity (80%) of the answer that the duration of vertigo was more than 1 min was calculated. It is important to note that central vestibular disorders might be the cause of vertigo longer than 1 min. Based on comparing the clinical picture of patients with PC-BPPV, HCG-BPPV and HCA-BPPV we established a diagnostic pattern useful in patients with vertigo. Patients with BPPV usually report: spin-like dizziness- vertigo, paroxysmal character of vertigo and attacks which are evoked by turning in bed. It is essential to remember to ask the patient how long the attacks last and what type of head movement evokes the attack. 

It is important to remember that vertigo attacks lasting more than minute might be also the symptom of the vertigo caused by central diseases.

## 4. Discussion

Despite increasing awareness of BPPV diagnosis and treatment, in our study 53.3% of patients had previously experience problems with BPPV but were not provided with accurate diagnosis and treatment.

The ability to accurately identify probable BPPV is time-saving as it facilitates effective management and appropriate referrals. However, it is important to emphasize that diagnostic maneuvers, such as the Dix-Hallpike maneuver and the supine roll test, remain essential for confirming a BPPV diagnosis. The paper presents a standardized questionnaire which can be helpful in distinguishing individual BPPV subtypes after performing appropriate diagnostic maneuvers.

Feeling dizzy while rolling over in bed is strongly linked to BPPV. According to the studies, the absence of vertigo when turning in bed allows BPPV to be excluded with 98% certainty [1]. Our results confirm that statement, in PC-BPPV and HCG-BPPV groups 100% of patients reported vertigo when turning in bed, in group HCA-BPPV it was 92.9% (13/14 patients). The second very characteristic symptom is short duration of vertigo. In the study groups more than 95% of patients reported that the duration of vertigo attacks was less than one minute. However, elderly patients are less likely to have typical nystagmus or spinning vertigo [15]. They more often report instability and loss of balance rather than typical vertigo. 

The mean age was 47.7 years among all study groups, which is the similar age to von Brevern et al. study [2]. The most frequently affected canal was the right posterior semicircular canal, which is the same result as previous studies [9,16].

In our research, we demonstrated that using responses of patients suffering from BPPV regarding head movement causing vertigo and the duration of the vertigo is effective for identifying the specific type of BPPV.

The movement of the head in the horizontal plane significantly more often evoked vertigo attacks in patients diagnosed with HCG-BPPV (75.0%). These patients also reported a significantly higher frequency of attacks than in other study groups (50.0% experienced attacks of vertigo more than once a day). The feeling of the undulation of the ground was observed more often in HCG-BPPV than in other groups. These observations result from the anatomy and pathophysiology HCG-BPPV. The horizontal canal is more sensitive to side-to-side head rotations, which are common movements throughout the day. When the otoliths move, they can trigger the more intense and prolonged sensation of spinning or vertigo each time the head is turned. Additionally, the horizontal canal is more responsive to gravity-induced changes when the body or head is in a horizontal position. When dislodged otoliths move more freely within the canal, they cause a stronger response from the vestibular system, resulting in more intensive vertigo and consequently nausea and vomiting. This is especially significant for active people who are unable to perform their professional duties because of BPPV. Furthermore, nausea and vomiting are associated with acute vestibular symptom (AVS), thus the clinical picture of HC-BPPV might mimic AVS. Diagnostic maneuvers for BPPV and the Head Impulse, Nystagmus, Test of Skew (HINTS) are crucial to make an accurate diagnosis. Nausea is often more prevalent in HCG-BPPV compared to PC-BPPV which is confirmed by the results. Nausea appeared in 63.4% of HCG-BPPV patients in comparison to 31.7% in PC-BPPV. The PC-BPPV typically results in episodes of vertigo with movements in the sagittal plane, such as looking up or bending over. These movements tend to be less frequent and less sustained than the continual left-right movements that affect the horizontal canal. Which is in line with the results that HCG-BPPV patients reported the biggest frequency of attacks of vertigo. Additionally, the horizontal canal has bigger cortical representation, which can lead to more intense response to irritation of horizontal canal sensory cells.

Interestingly, the presence of nystagmus during head-shaking test in patients with PC-BPPV and HC-BPPV was investigated by Lee and Kim [17,18]. They found that 48% of patients with HC-BPPV and 32% of patients with PC-BPPV had generated the head shaking nystagmus. The presence of head shaking nystagmus in HC-BPPV is probably a consequence that head shaking test is performed in axial plane. In the case of PC-BPPV authors explained that the movements of the otoliths associated with endolymph dynamics appear to play more crucial role than the velocity storage mechanism. These conclusions may explain why some patients with PC-BPPV experience vertigo during the head movement in axial plane.

One of the elements of the interview data analysis was the duration of the vertigo attack. According to literature data, in canalolithiasis the duration of triggered nystagmus is approximately 10 s (in rare cases up to 60 s) [14]. However, in the case of cupulolithiasis of the lateral semicircular canals, an attack of vertigo may last as long as the patient’s head is held in a critical position, which is caused by otoliths attached to cupula [19]. Moreover, in some cases, anxiety-driven patients may report a longer duration attack than it actually is [5]. Our results are in the line with the previous observations. Patients with HCA-BPPV reported significantly more often the duration of vertigo longer than one minute.

Patients diagnosed with PC-BPPV were the biggest group, they accounted for 60% of all people included in the study which is similar amount reported in the literature (60–86%) [3,4,9,20]. Vertigo evoked by movement of the sagittal plane itself was not as specific for the PC-BPPV group as might be expected, but the combination of short duration of vertigo and the induction of sagittal plane vertigo was significantly more sensitive and specific. 

One more interesting observation was that almost all patients with HCA-BPPV (92.9%) had vertigo evoked by movement of the head in sagittal plane and only 28.6% of them reported that movement of the head in axial plane caused vertigo. It may result from that horizontal canal is anteriorly uptilted approximately 30 degrees from the horizontal plane when the patient is in a sitting position, and the cupula is tilted in the sagittal plane with its base pointed medially [21]. Recently, important study has appeared about the diagnostic differentiation from HCA-BPPV and central disorders [22]. Patients with vertigo should be carefully evaluated neurologically, because apogeotropic nystagmus can be the symptom also in the central pathologies concerning cerebellum. 

The most common cause of BPPV is head trauma. In the BPPV patient population, a positive history of head trauma ranges from 8 to 23.4% of patients [23,24,25]. In this study, a history of head trauma was reported by 17 patients (26.9%) in the PC-BPPV group, 4 patients (14.3%) in the HCG-BPPV group and 5 patient (35.7%) in the HCA-BPPV group–a total of 24.8%. This is the higher percentage than in most publications. This may be due to the retrospective analysis based on questionnaires completed by patients. Additionally, there were no conditions regarding the severity of the head injury. Nevertheless, the nature and severity of injury that can cause BPPV varies considerably from minimal head trauma (being hit on the head with a ball during a game) through moderate to severe head and neck injuries with loss of consciousness [26]. There are no clear and accepted criteria for the diagnosis of post-traumatic BPPV in the scientific literature, nor is there a unified opinion on the time from injury to the onset of symptoms [27]. Regardless of whether the lesions were caused by trauma or not, the same maneuvers are used in the diagnosis and treatment of BPPV [13,27,28]. However, as patients with post-traumatic BPPV are more likely to have multi-canal BPPV, respond less well to treatment and have more frequent recurrences, that is why they need special attention in the diagnostic and treatment process. Patients who suffered from BPPV as a consequence being beaten may apply for compensation from the perpetrator.

The risk of BPPV is also increased in migraine [9,29,30,31]. According to Ishiayma et al., the incidence of migraine is three times higher in patients with idiopathic BPPV than in the general population. Moreover, patients with migraine had a higher recurrence of attacks (75%) after effectively performed repositioning maneuvers [32]. Ishiayma et al. report that 83% of patients with BPPV and migraine were women. According to the authors, this could explain the almost threefold higher incidence of BPPV in women in the general population [32]. The atrial arterial spasm in migraine patients is considered to be the etiological mechanism in otolith degeneration, and the recurrent vasospasms that occur in migraine are most likely responsible for the recurrence of BPPV attacks in this group [31,32,33]. In this study, the comorbidity of migraine and BPPV in study groups was found in a total of 20% patients of whom more than 80% were women. These results are in line with previous scientific work.

## 5. Conclusions

To conclude patients diagnosed with PC-BPPV usually had vertigo evoked by movement of the head in the sagittal plane and the duration of vertigo was usually less than 10 s. The attack of vertigo in patients with HCG-BPPV appeared after turning head side to side, the duration of symptoms was less than 1 min, the frequency of attacks was more than once a day and were accompanied by nausea and/or vomiting more often than in other subtypes of BPPV. The patients with HCA-BPPV suffered from attacks of vertigo much longer–more than one minute and less frequent than in other subtypes of BPPV.

## Figures and Tables

**Table 1:** The questionnaire.

				Yes	No
Do you get dizzy when laying down or turning over in bed?					
Do you get dizzy when looking up?					
Do you get dizzy when looking down?					
Are you unsteady by gait?					
Have you experienced previous head trauma?					
If yes, have the you had any health problems as a result of that head trauma ?					
Previous similar symptoms?					
Do you have neck problems?					
Do you suffer from any chronic diseases? If yes, please write them down ……………………………………					
Do you have spinning or a whirling sensation of the surroundings or yourself?					
For how long does the dizziness last?		A couple of seconds	Less than 1 min	More than 1 min	
How long does the problem with dizziness last?	1–7 days	1–4 weeks	1–6 months	6–12 months	more than 1 year

**Table 2:** The characteristics of symptoms during BPPV attack among all study groups.

	The Feeling of Undulation of the Ground	Nausea	Vomiting	The Frequency of Attacks (More than Once a Day)	The Duration of the Attacks (A Couple of Seconds)	Evoked Vertigo		
						By Turning in Bed	By Movement in Sagittal Plane	By Movement in Axial Plane
PC-BPPV	47.6%	31.7%	3.2%	34.9%	71.4%	100%	88.9%	15.9%
HCG-BPPV	82.1%	64.3%	32.1%	50.0%	50.0%	100%	100%	75.0%
HCA-BPPV	57.1%	14.3%	0.0%	14.3%	21.4%	92.9%	92.9%	28.6%
*p*-value	<0.01	<0.01	<0.01	<0.01	<0.01	0.04	>0.01	<0.01

The statistical analysis was performed using the modified chi-square test between three study groups with significance level *p* < 0.01. Legend: PC-BPPV—posterior canal benign paroxysmal positional vertigo; HCG-BPPV—horizontal canal benign paroxysmal positional vertigo with geotropic nystagmus; HCA-BPPV—horizontal canal benign paroxysmal positional vertigo with apogeotropic nystagmus.

**Table 3:** Relationship between the subtype of BPPV and combined answers to two questions: ‘What kind of head movement triggers vertigo?’ and ‘How long does the vertigo continue?’.

BPPV	Sagittal Plane, Couple of Seconds	Others	Total	Parameter	
**PC**	39	24	63	Sensitivity	69.6%
**HCA + HCG**	17	25	42	Specificity	51.0%
**Total**	56	49	105		
	**Axial plane, couple of seconds or <1 min**	**Others**	**Total**		
**HCG**	21	7	28	Sensitivity	61.8%
**PC + HCA**	13	44	77	Specificity	86.3%
**Total**	34	51	105		
	**>1 min**	**Others**	**Total**		
**HCA**	4	10	14	Sensitivity	80.0%
**PC + HCG**	1	90	91	Specificity	90.0%
**Total**	5	100	105		

Legend: PC—posterior canal benign paroxysmal positional vertigo; HCG—horizontal canal benign paroxysmal positional vertigo with geotropic nystagmus; HCA—horizontal canal benign paroxysmal positional vertigo with apogeotropic nystagmus.

## Data Availability

The original contributions presented in the study are included in the article, further inquiries can be directed to the corresponding author.
